# Determination of Cyclaniliprole in Fruits and Vegetables Using Disposable Pipette Extraction Cleanup and Ultrahigh-Performance Liquid Chromatography-Tandem Mass Spectrometry

**DOI:** 10.3390/molecules27196464

**Published:** 2022-09-30

**Authors:** Zhou Lu, Weiqian Yue, Weiming Ren, Yanhong Wang, Yueru Li

**Affiliations:** 1 Center of Quality Standard and Testing Technology for Agro-Products, Jilin Agricultural University, Changchun 130118, China; 2 College of Plant Protection, Jilin Agricultural University, Changchun 130118, China

**Keywords:** cyclaniliprole, diamide insecticide, residue analysis, DPX, UHPLC-MS/MS

## Abstract

Despite an outstanding agent for control of Lepidoptera, the diamide insecticide cyclaniliprole (CYCP) is a suspected carcinogen. In the present study, an analytical method was developed for the determination of CYCP in six fruits and vegetables (apple, grape, peach, bell pepper, lettuce, and tomato) using ultrahigh-performance liquid chromatography coupled with tandem mass spectrometry. Sample preparation was carried out by the acetonitrile-salting-out extraction followed by simple and fast cleanup of disposable pipette extraction tip containing styrene divinyl benzene and/or graphitized carbon black. Satisfactory linearity (*r* > 0.99) was obtained in the calibration range of 0.001–1 µg mL^−1^. Matrix effects decreased from −9.9–−17.9% to −1.0–−7.6% after the cleanup. The recoveries of CYCP at three spike levels (0.01, 0.1, and 1 mg kg^−1^) from different matrices were between 75.7% and 111.5%, with the intra-day (*n* = 5) and inter-day (*n* = 15) relative standard deviations lower than 12.1%. The limit of quantification was 0.01 mg kg^−1^. The developed method provides a good reference for routine monitoring of CYCP in these fruits and vegetables.

## 1. Introduction

**Figure 1:**
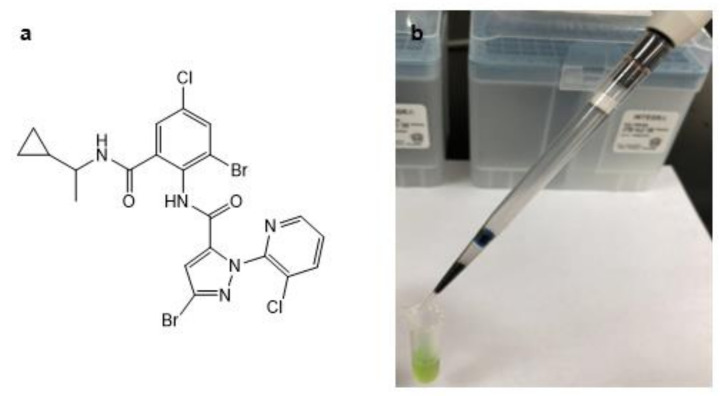
Molecular structure of CYCP (**a**) and a photograph of DPX apparatus (**b**).

Cyclaniliprole (CYCP; 2’,3-dibromo-4’-chloro-1-(3-chloro-2-pyridyl)-6’-{((1RS)-1-cyclopropylethyl)carbamoyl}pyrazole-5-carboxanilide) is a newly developed insecticide introduced by Ishihara Sangyo Kaisha, Ltd. (Osaka, Japan) [1]. Its chemical structure is shown in Figure 1a. CYCP belongs to anthranilic diamides, and members of this class act by binding to insect ryanodine receptors, leading to lethargy, paralysis, and death due to the unregulated loss of intracellular calcium stores [2]. CYCP effectively controls major agricultural Lepidoptera such as *Plutella xylostella*, *Mythimna separata*, and *Spodoptera litura* in a wide range of crops. Unlike other diamide compounds such as chlorantraniliprole and cyantraniliprole, the developer claimed that CYCP has a structural advantage that makes it more active against diamide-resistant insects [3]. As the resistance of pests to early diamides such as chlorantraniliprole has been continuously identified [4,5,6], CYCP could be an important complement to this insecticide class to overcome this issue. 

The substantial contribution made by pesticides to agriculture is obvious. At least a third of our crop production will be wasted due to damage by various pests and pathogens if there are no pesticides applied [7]. However, the unreasonable use of pesticides will exhibit adverse effects on food safety and further endanger human health. Although CYCP has low mammalian toxicity with the oral median lethal dose (LD_50_) to rats > 2000 mg/kg b.w., it is suspected to be a carcinogen, as it induces C-cell adenoma in male rats according to the report from the European Food Safety Authority [8]. To avoid this risk, governments have established laws or regulations to specify the maximum residue limits (MRLs). Residue analysis methods are essential means to ensure the pesticide residue level in foods is in compliance with MRLs. 

Pretreatment is necessary for raw samples to transform into a certain status amenable to instrumental analysis. The Quick, Easy, Cheap, Rugged, and Safe (QuEChERS) method has been widely used in this process. Developed by Anastassiades et al. [9], QuEChERS extracts samples with acetonitrile (MeCN) and then partitions the aqueous/organic phases by adding MgSO_4_ and NaCl. The MeCN extract is cleaned up by thoroughly mixing it with the adsorbent primary secondary amine to remove interfering co-extractives, which is called dispersive-solid phase extraction (d-SPE). Soon afterward, this methodology evolved into the buffering versions effectively dealing with some pH-sensitive pesticides [10,11], which finally led to two official methods, AOAC 2007.01 [12] and EN 15662 [13], depending on whether the acetate or the citrate buffering strategy was used. Nowadays, the QuEChERS is still an open source system as researchers continue to modify it to achieve higher efficiency in the extraction and cleanup of various types of samples [14,15,16,17,18]. 

Disposable pipette extraction (DPX) is a novel cleanup technique developed to incorporate d-SPE and solid-phase extraction (SPE) approaches. A photograph of the DPX apparatus is shown in Figure 1b. The efficiency of DPX cleanup is mainly attributed to the repeated d-SPE process that occurs when the sample extract is aspirated and dispensed through the pipette tip and fully contacts the freely moving adsorbents packed in it [19]. Furthermore, after mixing, adsorbents with higher weight will be quickly deposited at the end of the tip to form a “micro-SPE” layer so that when the liquid is dispensed, the samples undergo another cleanup step as they pass through the deposit adsorbent. The DPX technique also saves more time as the centrifugation required in the d-SPE process is not necessary here. So far, DPX has been successfully applied for the analysis of various organic contaminants in food and environmental samples [20,21,22,23,24]. 

The present study developed and validated an analytical method for the determination of a newly developed anthranilic diamide insecticide CYCP in fruits and vegetables using ultrahigh-performance liquid chromatography coupled with tandem mass spectrometry (UHPLC-MS/MS). Samples were extracted by MeCN-salting-out, and the extract was cleaned up using DPX tips packed with styrene divinyl benzene (SDVB) and/or graphitized carbon black (GCB). The effects of different adsorbents and instrumental conditions on the method performance were investigated. Real sample analysis was conducted as the application of the developed method. 

## 2. Results and Discussion

### 2.1. MS/MS Optimization

MS/MS was run in both positive (ESI+) and negative (ESI−) modes to identify the appropriate CYCP precursor ion. As a result, the [CYCP + H]^+^ of 599.9 Da was identified under ESI+ (Figure S1a), while [CYCP − H]^−^ of 597.9 Da appeared under ESI—(Figure S1c). Afterwards, certain energy was provided for each precursor ion to fragment into product ions. As shown in the MS2 spectrum of ESI+ (Figure S1b), two product ions (283.8 Da and 514.8 Da) with reasonable intensities were observed. By comparison, only one product ion (256.1 Da) with an acceptable response was observed in that of ESI− (Figure S1d). Since MS analysis of residues usually requires the identification of an analyte by at least two characteristic product ions, ESI+ mode was used for the detection of CYCP in our method, and the product ion of 283.8 Da, which has a relatively high intensity, served as the quantifier. 

A number of MS/MS parameters that could affect the signal strength of ion transitions including declustering potential (DP), collision energy (CE), and collision cell exit potential (CXP) were optimized by running a ramp over a certain range, and the value that yielded the highest response was adopted. 

### 2.2. Chromatography Optimization

**Figure 2:**
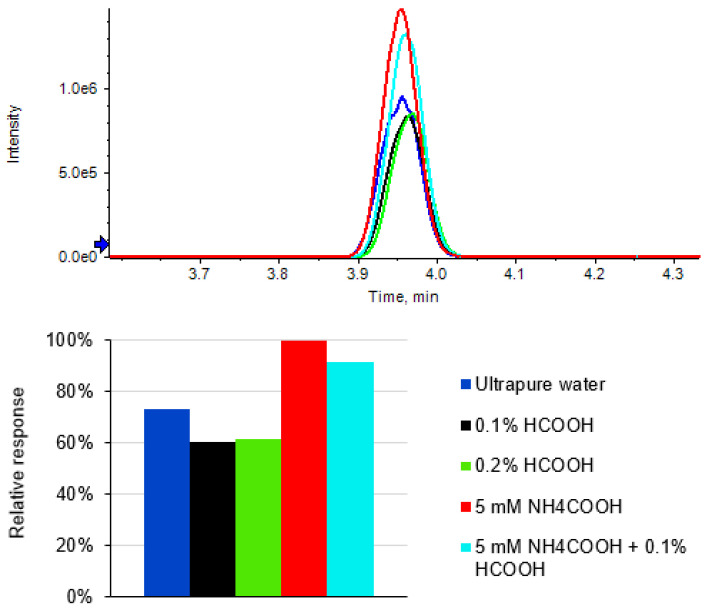
Effects of different LC aqueous phases on peak shape and response of CYCP.

The addition of some volatile acids or salts in the LC mobile phase could affect either the peak shape or signal response of the analyte during an LC-MS analysis [25,26]. The shape of a chromatographic peak is important for its integration, leading to accurate quantification, and an increased response can improve the detection sensitivity. In this study, we tested five different aqueous-phase (solvent A) compositions (a. ultrapure water; b. 0.1% HCOOH; c. 0.2% HCOOH; d. 5 mM NH_4_COOH; and e. 5 mM NH_4_COOH + 0.1% HCOOH) to determine the one with the best performance. As shown in Figure 2, by comparison with ultrapure water, the addition of HCOOH suppressed the signal, while the LC-MS provided both good peak shape and the highest response for CYCP when 5 mM NH_4_COOH was used as the additive in the LC aqueous phase. Therefore, the use of composition d was adopted by the developed method. 

### 2.3. Sample Preparation Optimization

**Figure 3:**
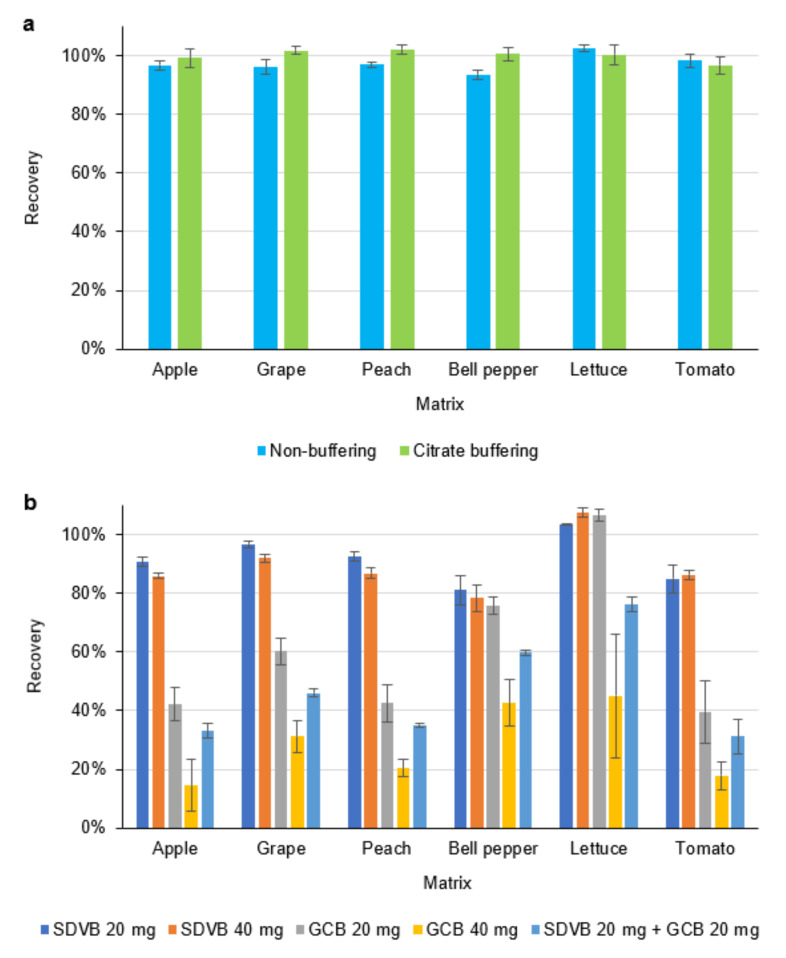
Effects of different extraction (**a**) and cleanup (**b**) strategies on recoveries of CYCP from fruit and vegetable samples.

The present study compared the efficiency of the citrate (Na_3_Citrate/Na_2_HCitr) buffering and non-buffering extraction method and the results are shown in Figure 3a. CYCP recoveries from six matrices extracted by both approaches were in the satisfactory range of 93.6–102.5%, while no significant difference in recoveries from each individual matrix was observed. This indicated that the stability of CYCP could be maintained in matrices with different pH values. Therefore, from an economical point of view, non-buffering extraction method was adopted by the developed method. 

SDVB is good at removing non-polar and weakly polar co-extractives in agricultural products such as lipids, waxes, and steroids [27], while GCB is commonly used for adsorbing pigments [28]. In this study, the cleanup performance of DPX tips containing five sets of adsorbents (a. 20 mg of SDVB; b. 40 mg of SDVB; c. 20 mg of GCB; d. 40 mg of GCB; and e. 20 mg of SDVB + 20 mg of GCB) was investigated. Anastassiades and Lehotay proposed an empirical rule according to a number of previous studies on d-SPE cleanup: every milliliter of sample extract combined with 50 mg of adsorbent provides satisfactory recoveries with a wide analyte scope [29]. As we have decided that 800 µL of extract is adequate for both in-tip mixing and instrumental analysis, the appropriate amount of adsorbent according to this rule would be 40 mg. Due to the limited space in the tip, the performance of less adsorbent use (20 mg) was also investigated. 

The results (Figure 3b) showed that the use of SDVB of 20 and 40 mg yielded similar CYCP recoveries within a satisfactory range (78.3–107.4%) for all matrices. However, the use of GCB substantially reduced the recoveries of CYCP to 14.5–60.1% for apple, grape, peach, and tomato. This influence was attenuated for bell pepper and lettuce as the recoveries increased to 42.6–106.6%. Notably, when 20 mg of GCB was employed, acceptable recoveries (75.7% for bell pepper and 106.6% for lettuce) were obtained for these two green vegetables. We could extrapolate two possible reasons accounting for this: first, as apple, grape, peach, and tomato contain more organic acids than lettuce and bell pepper, the lower pH values of their extracts may facilitate the adsorption of CYCP on GCB; second, when the d-SPE process occurred between GCB and the matrices of lettuce and bell pepper, the carbon material preferentially adsorbed certain pigments such as chlorophyll, leading to higher CYCP recoveries. 

**Figure 4:**
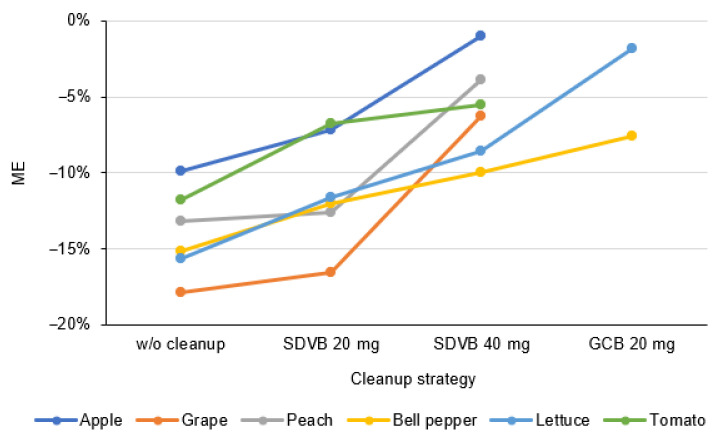
Effects of different cleanup strategies on the ME of fruit and vegetable samples.

Since a number of adsorbent sets provided good and similar recoveries for one specific matrix, we further compared their co-extractive removal ability with respect to ME reduction. As shown in Figure 4, 40 mg of SDVB reduced ME to the minimum for apple, grape, peach, and tomato, while 20 mg of GCB led to the lowest ME for bell pepper and lettuce. As a result, DPX tips containing 40 mg of SDVB were used for the cleanup of apple, grape, peach, and tomato extracts, and tips containing 20 mg of GCB were employed for the purification of bell pepper and lettuce extracts. 

### 2.4. Method Validation

**Figure 5:**
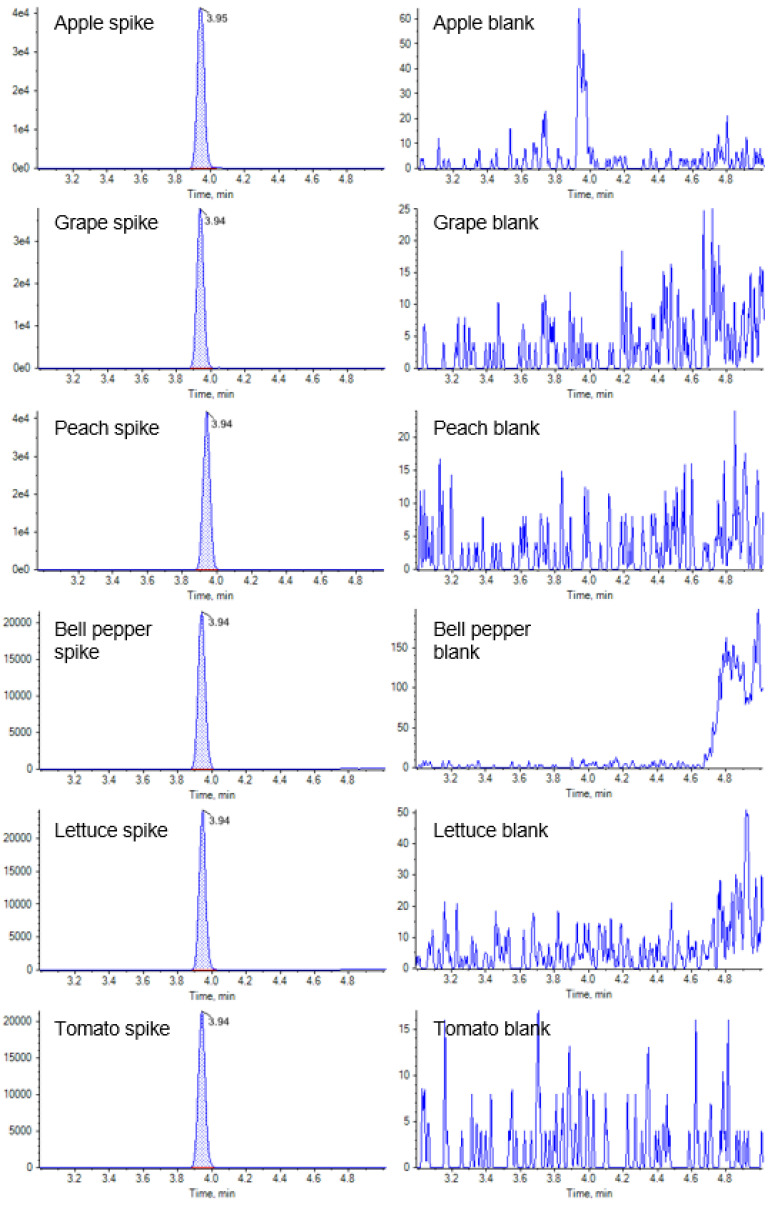
Typical MRM chromatograms of spiked (0.01 mg kg^−1^) (left-hand column) and blank samples (right-hand column).

Typical MRM chromatograms of spiked and blank samples are shown in Figure 5. No interference from the blank matrices appeared at the retention time of CYCP. 

Information on calibration, ME, LOQs, and regulated MRLs of CYCP in different matrices is listed in Table 1. Good linearity (*r* > 0.99) was achieved for each matrix-matched calibration curve in the range of 0.001–1 µg mL^−1^. MEs with positive and negative values correspond to signal enhancement and suppression, respectively. In this study, all matrices exhibited suppression to CYCP response, and ME decreased from −9.9–−17.9% to −1–−7.6% after DPX cleanup. 

Mean recoveries of CYCP from the six fruits and vegetables that were spiked at 0.01, 0.1, and 1 mg kg^−1^ were in the range of 75.7–111.5%, and the corresponding intra-day (*n* = 5) and inter-day (*n* = 15) relative standard deviations (RSDs) ranged from 0.4% to 12.1%. The results conformed to the method performance acceptability criteria (70% ≤ recovery ≤ 120%; RSD ≤ 20%) required by SANTE [30]. Detailed data are shown in Table 2. The LOQs was 0.01 mg kg^−1^ in all matrices according to SANTE guidelines [30], which were lower than relevant MRLs regulated by USA and identical to those regulated by EU.

CYCP was not detected (> LOQ) in all collected real samples (10 for each matrix, 60 in total). As CYCP has not been registered for use in China [31], this result is thought to be rational.

## 3. Materials and Methods

### 3.1. Chemicals and Reagents

The analytical standard of CYCP (purity 99.0%) was provided by Shenyang Research Institute of Chemical Industry (Shenyang, China). Anhydrous MgSO_4_, NaCl, sodium citrate tribasic dehydrate (Na_3_Citrate), and sodium citrate dibasic sesquihydrate (Na_2_HCitr) were purchased from Agilent Technologies (Palo Alto, CA, USA). LCMS-grade MeCN and formic acid (HCOOH) were obtained from DiKMA Technologies (Beijing, China). Ammonium formate (NH_4_COOH; purity ≥ 99.995%) was purchased from Sigma-Aldrich (St. Louis, MO, USA). DPX tips (1250 µL) containing different amounts of SDVB and/or GCB were bought from DPX Technologies (Columbia, SC, USA). A manual pipette (100–1000 µL) used for DPX cleanup was obtained from INTEGRA Biosciences (Zizers, Switzerland). The water used in this study was ultrapure (18 MΩ cm) and was prepared by a LAB-UV-40 water purification system manufactured by Lab-Partner Technology Development (Changchun, China). 

A 0.01 g sample of CYCP standard was dissolved in 10 mL of MeCN to prepare the stock solution at 1000 µg mL^−1^. The solution was stored in an amber vial in a refrigerator at 4 °C, and its stability in three months was guaranteed by UHPLC-MS/MS monitoring. Working solutions with lower concentrations were freshly prepared from the stock solution before their use. 

### 3.2. Sample Preparation

Samples of apple, grape, peach, bell pepper, lettuce, and tomato were bought from a local agro-product market and pureed in a food processor (Braun, Kronberg, Germany). For sample extraction, 10.0 g (±0.1 g) of a certain processed fruit or vegetable sample was taken into a centrifuge tube (50 mL) and then added to 10.0 mL of MeCN. The tube was capped and vigorously hand-shaken for 1 min. Afterward, 1 g of NaCl and 4 g of anhydrous MgSO_4_ were added. The tube was shaken again for 30 s and then centrifuged at 5000 rpm for 5 min. Finally, 800 µL of the supernatant was transferred to a centrifuge tube (2 mL) waiting for DPX cleanup. 

The manual pipette equipped with the DPX tips containing 40 mg of SDVB was used for cleanup of extracts of apple, grape, peach, and tomato, while the tips containing 20 mg of GCB were employed for purifying those of bell pepper and lettuce. Aspirating volume of the pipette was set to be 1000 µL, and the extract supernatant was aspirated slowly in and out of the DPX tip three times before it was dispensed into a 2 mL vial for UHPLC-MS/MS analysis. 

### 3.3. Instrumentation

A Nexera UHPLC system (Shimadzu, Kyoto, Japan) coupled to a QTRAP4500 MS/MS (Sciex, Framingham, USA) was used for the detection of CYCP. The hybrid system was controlled by Analyst 1.6.2 software (Sciex, Framingham, USA). The UHPLC was equipped with a Luna Omega 2.1 × 100 mm, 1.6 µm C_18_ column (Phenomenex, Torrance, USA) held at 40 °C. The mobile phase comprised 5 mM NH_4_COOH (solvent A) and MeCN (solvent B) with a constant flow rate of 0.3 mL min^−1^. The gradient elution used was 45%B (0.0 min) → 95%B (6.0 min) → 95%B (7 min) → 45%B (7.1 min) → 45%B (10.0 min). The injection volume was 5 µL. 

The MS/MS was equipped with an electrospray ionization (ESI) source operating in the positive mode (ESI+). Qualification of CYCP was fulfilled by two ion transitions under multiple reaction monitoring (MRM) mode, and the one with the highest abundance was selected for quantification. Relevant MRM parameters are shown in Table 3. Other conditions used were as follows: ionspray voltage, 5500 V; source temperature, 550 °C; curtain gas pressure, 0.2 mPa; ion spray gas pressure, 0.3 mPa; auxiliary heating gas pressure, 0.3 mPa; dwell time for each transition, 100 ms. MultiQuant 3.0.3 software (Sciex, Framingham, USA) was employed for data analysis. 

### 3.4. Method Validation

CYCP content in different matrices was quantified by the external standard method. Since the influence of matrix effects (ME) on the quantification is inevitable during LC-MS analysis [32], a 7-point (0.001, 0.005, 0.01, 0.05, 0.1, 0.5, and 1 µg mL^−1^) matrix-matched calibration curve instead of a curve comprising solvent standards was used to deal with any signal enhancement or suppression caused by ME. The extent of the ME on analyte ion abundance in different matrices was measured by the following equation [33]:

$$\mathrm{ME}=(\frac{\mathrm{Slope}\;\mathrm{of}\;\mathrm{matrix}-\mathrm{matched}\;\mathrm{calibration}\;\mathrm{curve}}{\mathrm{Slope}\;\mathrm{of}\;\mathrm{solvent}\;\mathrm{calibration}\;\mathrm{curve}}-1)\times 100\%$$

The developed method was validated with respect to recovery (accuracy), and corresponding relative standard deviations (RSDs) (precision) by spiking blank samples at three levels of 0.01, 0.1, and 1 mg kg^−1^. The recovery test at each spike level was conducted as intra-day (*n* = 5) and inter-day (*n* = 15). Based on SANTE/11312/2022 guidelines [30], the limit of quantification (LOQ) is identified as the lowest spike level of the analyte in matrix. 

Ten samples of each kind of fruit and vegetables used in this study were purchased from 5 food markets in Changchun, Jilin, China. All collected samples (*n* = 60) were pretreated and analyzed using the developed method to assess the residue level of CYCP and further validate the reliability of this method. 

## 4. Conclusions

The present study combined DPX sample cleanup with UHPLC-MS/MS detection to provide rapid and accurate determination of CYCP in six fruits and vegetables. Despite its excellent Lepidopteran control efficacy, CYCP is a possible carcinogen, so its contents in agricultural products must be monitored routinely to avoid any potential risks caused by its inappropriate use. The performance of this method satisfies relevant requirements of the EU analytical quality control regulation SANTE; thus, it can serve as a fast and reliable approach fulfilling the above purposes. 

## Figures and Tables

**Table 1:** Information on calibration, ME, LOQs, and MRLs of CYCP in different matrices.

Matrix	Calibration Equation	r	ME (%)	LOQ (mg kg^−1^)	MRL (mg kg^−1^) (EU/US)
Acetonitrile	y = 6472x − 148	0.9987	-	0.01	-
Apple	y = 6407x + 384	0.9980	−1.0	0.01	0.01/0.3
Grape	y = 6062x + 335	0.9947	−6.3	0.01	0.01/0.8
Peach	y = 6218x + 923	0.9934	−3.9	0.01	0.01/1
Bell pepper	y = 5980x + 347	0.9972	−7.6	0.01	0.01/0.2
Lettuce	y = 6356x + 965	0.9921	−1.8	0.01	0.01/15
Tomato	y = 6118x + 291	0.9974	−5.5	0.01	0.01/0.2

**Table 2:** Recoveries, RSD_a_ (intra-day, *n* = 5), and RSD_r_ (inter-day, *n* = 15) of CYCP from different fruit and vegetable samples analyzed by the developed method.

Matrix	Spike Level (mg kg^−1^)	Day 1		Day 2		Day 3		RSD_r_ (%)
		Rec. (%)	RSD_a_ (%)	Rec. (%)	RSD_a_ (%)	Rec. (%)	RSD_a_ (%)	
Apple	0.01	95.2	5.1	98.2	4.0	97.3	7.9	5.6
	0.1	85.8	0.9	102.6	1.7	102.1	1.3	7.5
	1	93.6	1.8	97.9	2.6	97.6	3.7	3.4
Grape	0.01	91.2	2.7	98.0	4.9	98.6	4.2	5.2
	0.1	91.9	1.3	107.5	0.8	105.8	0.4	6.4
	1	90.5	2.4	98.1	2.2	98.3	1.2	4.3
Peach	0.01	90.9	3.0	100.4	1.7	102.1	1.6	5.6
	0.1	86.7	1.9	110.8	4.5	102.7	2.6	9.9
	1	93.1	2.2	98.4	1.5	99.1	1.1	3.2
Bell pepper	0.01	86.2	2.0	102.2	0.8	101.1	1.9	7.9
	0.1	75.7	2.8	102.9	1.4	101.0	1.4	12.1
	1	88.2	1.6	102.3	1.1	103.8	0.5	7.5
Lettuce	0.01	88.4	2.3	108.4	1.0	107.2	2.1	9.5
	0.1	106.6	2.0	111.5	1.4	110.0	1.5	2.3
	1	88.7	2.3	105.2	0.9	106.3	0.9	8.4
Tomato	0.01	83.8	3.3	95.9	2.1	95.9	4.0	7.1
	0.1	86.2	1.7	100.4	1.5	100.0	1.7	6.5
	1	90.4	1.8	94.5	1.5	94.9	2.0	2.8

**Table 3:** MS/MS parameters for detection of CYCP.

Compound	Molecular Formula	Retention Time (min)	Ion Transition (m/z)	DP (V)	CE (V)	CXP (V)	MRM Ratio
CYCP	C_21_H_17_Br_2_Cl_2_N_5_O_2_	3.94	599.9 > 283.8 ^a,b^; 599d.9 > 514.8 ^a^	90; 90	23; 32	9; 21	0.28

^a^ For qualification, ^b^ For quantification.

## Data Availability

Not applicable.

## Supplementary Materials

The following supporting information can be downloaded at: https://www.mdpi.com/article/10.3390/molecules27196464/s1, Figure S1: Spectrum of CYCP using Q1 ESI+ scan (mass range 560–630 Da) (a). Spectrum of product ions of selected CYCP parent ion at 599.9 Da using ESI+ product ion mode (mass range 50–610 Da) (b). Spectrum of CYCP using Q1 ESI− scan (mass range 560–630 Da) (c). Spectrum of product ions of selected CYCP parent ion at 599.9 Da using ESI− product ion mode (mass range 50–610 Da) (d).

## Sample Availability

Samples of the compounds are not available from the authors.
