# Supplementary material for: Determination of Cyclaniliprole in Fruits and Vegetables Using Disposable Pipette Extraction Cleanup and Ultrahigh-Performance Liquid Chromatography-Tandem Mass Spectrometry
Source: Molecules. 2022 Sep 30;27(19):6464. doi: 10.3390/molecules27196464 (PMC9570770; doi:10.3390/molecules27196464)
Supplement: Supplementary file 1 [file molecules-27-06464-s001.zip › molecules-1921674-supplementary.pdf]

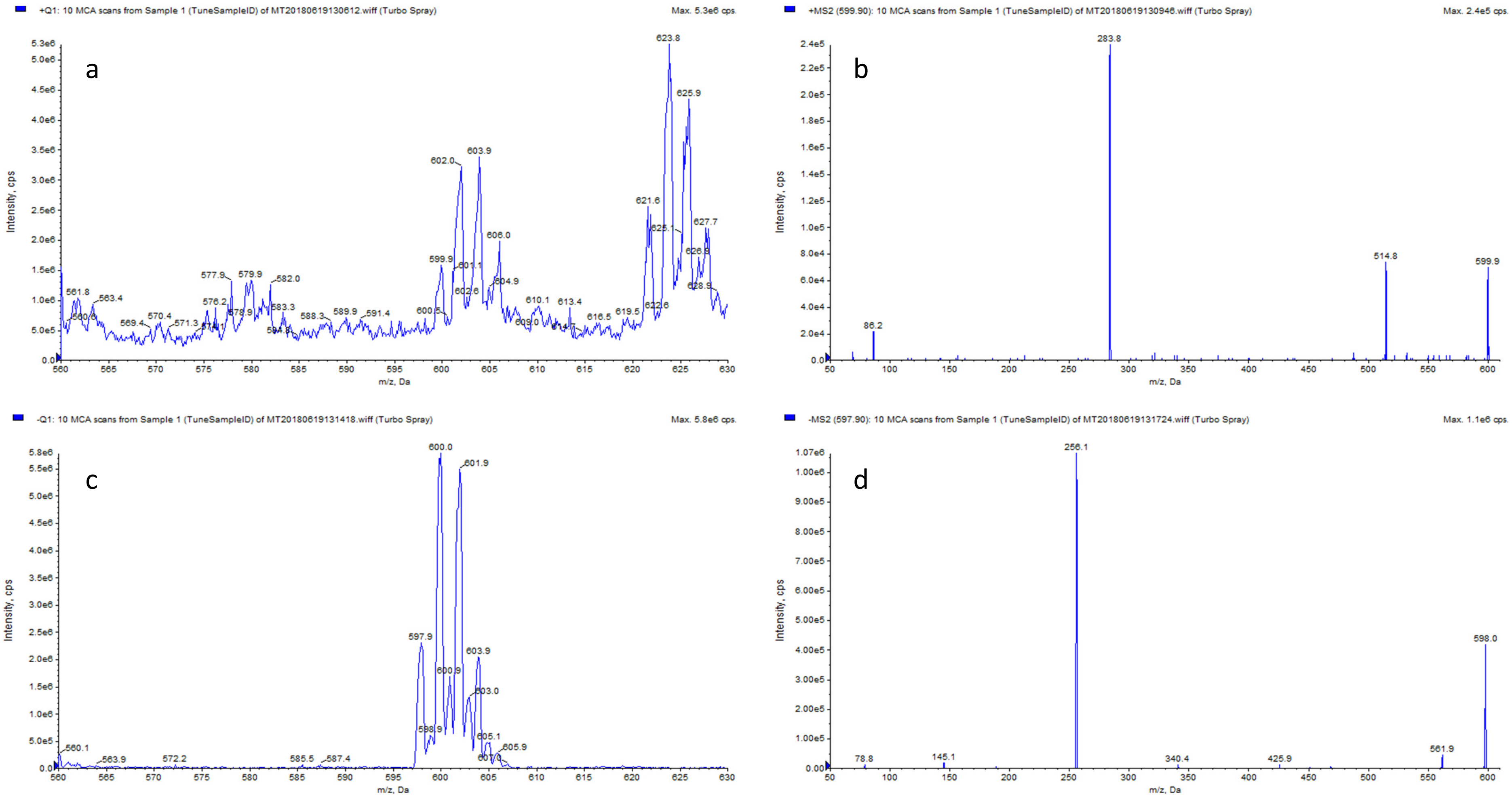

Figure S1. Spectrum of CYCP using Q1 ESI+ scan (mass range 560-630 Da) (a). Spectrum of product ions of selected CYCP parent ion at 599.9

Da using ESI+ product ion mode (mass range 50-610 Da) (b). Spectrum of CYCP using Q1 ESI− scan (mass range 560-630 Da) (c). Spectrum of product ions of selected CYCP parent ion at 599.9 Da using ESI− product ion mode (mass range 50-610 Da) (d).
